# Multi-Layer Workpieces and Multiple-Wire Electrochemical Micromachining with Horizontal Electrolyte Flushing

**DOI:** 10.3390/mi16111236

**Published:** 2025-10-30

**Authors:** Xiaocong Tang, Yongbin Zeng

**Affiliations:** 1 College of Mechanical and Electrical Engineering, Nanjing University of Aeronautics and Astronautics, Nanjing 210016, China; txcsq90@nuaa.edu.cn; 2 Nanjing Institute of Measurement and Testing Technology, Nanjing 210049, China

**Keywords:** multi-layer workpieces and multiple wires, wire electrochemical machining, horizontal electrolyte flushing, flow-field simulation, arrayed microstructures, machining efficiency

## Abstract

The multi-layer workpiece and multi-wire electrochemical microfabrication method (MWECM) has considerable potential in improving production efficiency and is considered a promising technology for manufacturing high-quality array microstructures. However, due to the accumulation of electrolytic by-products between workpiece layers, the machining accuracy is relatively low, which still limits its application in industrial environments. To address this issue, this article introduces a method to enhance mass transfer, which involves multi-layer workpieces and multi-wire electrochemical microfabrication, and employs horizontal electrolyte flushing (MWECMF). This innovation promotes the effective discharge of electrolytic deposits, thereby enhancing the renewal of electrolytes within the electrode gap. And use flow field simulation to optimize the interlayer spacing of workpieces and determine the optimal workpiece spacing. In addition, single factor experiments were conducted to determine the optimal processing parameters, including wire feed speed, power supply voltage, frequency, and duty cycle. Finally, at a feed rate of 1.2 µm/s, an array microstructure was successfully fabricated using a two-wire electrode setup and a four-layer workpiece configuration, achieving an overall machining rate of 9.6 µm/s. Compared to traditional tools or workpiece vibration mass transfer, the MWECMF method significantly improves the machining efficiency of wire electrochemical microfabrication (WECM).

## 1. Introduction

Wire electrochemical micromachining (WECM) is an advanced electrochemical micromachining technique designed for fabricating microscale components through the theory of electrochemical anode dissolution [1,2]. During the process, the wire operates without contact with the workpiece, thereby eliminating tool wear, preventing workpiece deformation, and avoiding the development of a heat-affected zone [3,4]. By utilizing a wire as the cathode, WECM circumvents the need for complex-shaped electrode fabrication, shortens pre-processing preparation time, and reduces production costs [5]. Especially in difficult-to-process materials, high surface quality can be achieved, which can be used for polishing and has better advantages compared to mechanical polishing. However, it cannot polish non-conductive materials [6,7,8]. The electrolyte used in micro electrochemical wire cutting can be classified into acidic, neutral, and alkaline based on its pH value. Acidic and neutral electrolytes are commonly used when processing stainless steel materials, such as HCl, anhydrous citric acid [9], NaNO_3_ [10], NaCl electrolytes, etc. Neutral electrolytes are more environmentally friendly, while the processing accuracy of sodium nitrate solution is higher than that of sodium chloride solution. Moreover, the wire electrode offers significant advantages for machining high-aspect-ratio structures [11]. Since material removal occurs at the ionic level, WECM is theoretically capable of producing microfeatures at micron or even nanometric scales by adjusting appropriate process parameters. Nevertheless, the narrow machining gap in WECM hinders the effective evacuation of electrolytic by-products, leading to slow electrolyte refreshment and low machining efficiency [12], which currently limits its broad industrial adoption.

To enhance machining efficiency and improve electrolyte renewal within the machining gap, numerous experimental studies have been conducted internationally in recent years. Regarding power supply modalities, the application of bipolar pulsed voltage has been shown to mitigate the accumulation of electrolysis by-products on the electrode surface. This reduction contributes to improved mass transfer rates and higher precision in machining processes [13]. Alternative strategies include the use of jet electrolyte flow introduced by El-Taweel et al. [14] and axial electrolyte flushing implemented by Qu et al. [15]. These methods enhance the elimination of electrolysis byproducts and improve electrolyte renewal. Additionally, reciprocating motion of the wire electrode along the axial direction has been proposed to augment mass transport and renew the electrolyte within the gap. Further studies indicate that modifying the wire electrode surface with microstructures or altering its geometry [16,17,18] can also improve mass transfer and machining quality during reciprocating operations. Meng et al. [19] reported the use of axial vibration multi-wire electrodes to improve the machining efficiency of array microstructure WECMM. Processing micro patterns on 100 µm thick nickel based metallic glass using tungsten wire with a diameter of 10 µm, with a speed of up to 0.8 µm/s. Zhang et al. [20] used transverse flushing to process stainless steel, and the results showed that transverse flushing can remove the electrolytic products on the upper and lower surfaces of the workpiece and update the electrolyte, improving mass transfer efficiency. In addition, Qu et al. [21] found that when using ethylene glycol solution to process 100 µm thick 3J21 alloy, bubble chains are generated. As the bubbles rise, they drive the electrolyte in the processing area to move upward, thereby promoting the discharge of electrolytic products. They successfully prepared a complex microstructure with a surface roughness Ra of 0.025 µm, but the feed rate is only 0.2 µm/s. The maximum feed rate can reach 1.2 µm/s. While these approaches contribute to enhanced mass transfer to varying degrees, their overall impact on machining efficiency remains limited.

MWECM is an emerging technology that extends conventional WECM through the use of arrayed micro-tools. By employing multiple wire electrodes to simultaneously machine layered stack structures, MWECM significantly increases throughput. This approach is particularly advantageous for fabricating array microstructures. However, experimental evidence indicates that during MWECM, substantial accumulation of electrolysis by-products—especially hydrogen bubbles—between the layers obstructs electrolyte renewal, thereby limiting the achievable electrochemical machining rate. This paper proposes a lateral flushing-assisted multi-wire electrochemical micromachining method for layered workpieces, which promotes the timely removal of electrolysis products trapped between layers, enables effective electrolyte refreshment within the machining gap, and improves overall process efficiency. Numerical simulations of the flow field are conducted to optimize inter-layer workpiece spacing, and experimental studies are carried out to determine optimal processing parameters.

## 2. Principle of MWECMF and Experimental System

**Figure 1:**
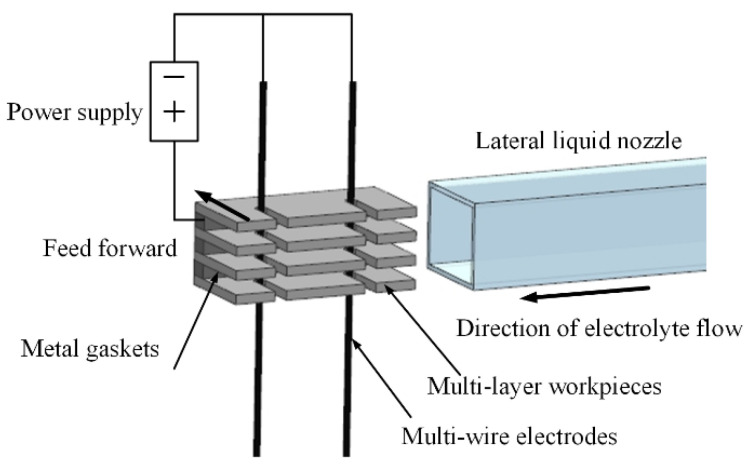
Schematic diagram of MWECMF.

**Figure 2:**
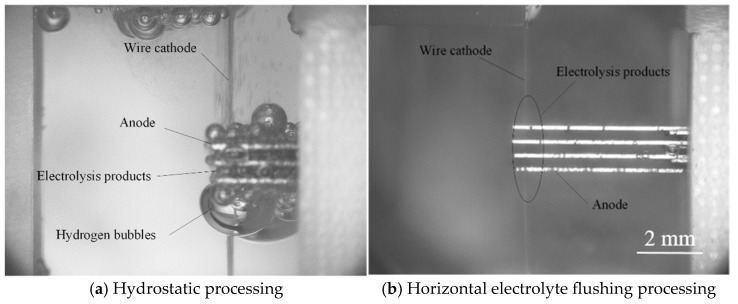
Comparison of effects before and after flushing.

Figure 1 depicts the schematic of the MWECMF system. In this configuration, the multilayer workpiece is coupled to the anode of the pulse power supply, and multiple wire electrodes, arranged in parallel, are connected to the cathode. Micro amplitude vibration enhanced mass transfer using wire electrodes. A relatively straightforward computer numerical control (CNC) motion strategy is employed to accomplish the machining objective. The nozzle is a 10 × 10 mm square, and the flow rate is calculated by measuring the liquid output per unit time. The filter element level in the filtration system is 1 µm, which can effectively filter insoluble substances in the electrolytic product. The electrolyte circulation system can monitor the temperature and conductivity of the electrolyte in real time, making it convenient to adjust the temperature and conductivity. The rising and falling edges of the high-frequency pulse power supply are 2 µs. Simultaneously, electrolyte solution is injected by a lateral flushing system and flows at a controlled rate between the workpiece layers, effectively removing accumulated by-products—particularly hydrogen bubbles—from the inter-layer zones. A comparison of the machining effects with and without flushing is presented in Figure 2. Figure 2a illustrates the outcome under static electrolyte conditions. While some bubbles escape the machining zone due to buoyancy, a significant portion accumulates within the inter-layer gaps, impairing mass transport efficiency and adversely affecting both machining accuracy and process efficiency. In contrast, Figure 2b demonstrates the result with lateral electrolyte flushing, where bubbles are promptly evacuated from the machining area, preventing any accumulation between workpiece layers. As a result, electrolytic by-products are efficiently expelled into the intermediate region and removed from the machining zone via cross-flow flushing. This continuous electrolyte refreshment promotes processing stability and enhances overall machining efficiency.

**Figure 3:**
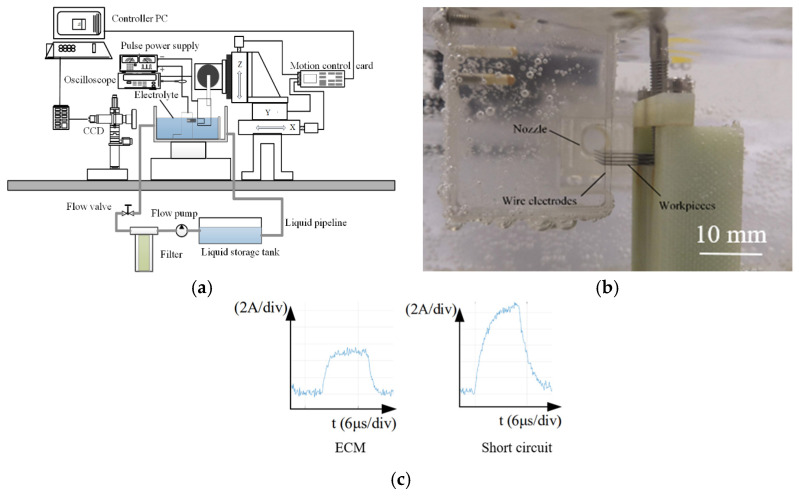
Introduction to the MWECMF system. (**a**) Schematic diagram of MWECMF system; (**b**) Physical image of processing area; (**c**) Typical current waveform during MWECMF.

A schematic diagram of the MWECMF system’s architecture is presented in Figure 3. Key components include the wire electrode assembly, multi-layer workpiece setup, pulse power supply, CNC feed system, CCD-based vision aid system, oscilloscope, and lateral flushing system. The visual monitoring system equipped with a CCD camera allows for real-time observation of the machining state, while the oscilloscope is employed to track the machining current. Figure 3c shows a typical current waveform during the electrochemical machining process. The left figure shows the normal waveform, and the right figure shows the waveform during a short circuit. The current value increases rapidly, so it is possible to determine whether the machining is normal based on the waveform. Within the flushing system, a pump circulates the electrolyte through a filtration unit before delivering it at a predetermined flow rate from a flushing nozzle into the inter-layer regions. This flow effectively eliminates electrolysis products and ensures continuous renewal of the electrolyte between the workpieces.

## 3. Results

### 3.1. Flow Field Simulation Research

**Figure 4:**
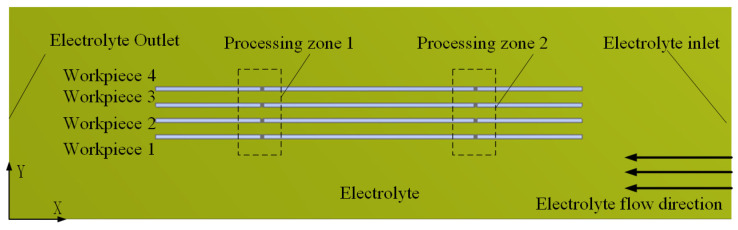
The flow field simulation model of MWECMF.

The distance between the electrodes dictates the resultant electrolyte flow rate. A low interlayer electrolyte flow rate makes it difficult to remove accumulated gaseous by-products. Moreover, it is essential to maintain uniform flow velocities across different layers. Therefore, flow field simulations were conducted using ANSYS Fluent 15.0 to evaluate various workpiece spacing configurations, thereby facilitating the selection of an appropriate gap for experimental validation. The electrolyte flow behavior was examined under different spacer thicknesses. A two-dimensional simulation model, as illustrated in Figure 4, was constructed with dimensions of 44 mm in length and 10 mm in width. The setup involves two wire electrodes and a four-layer workpiece stack. Detailed model parameters are provided in Table 1.

**Figure 5:**
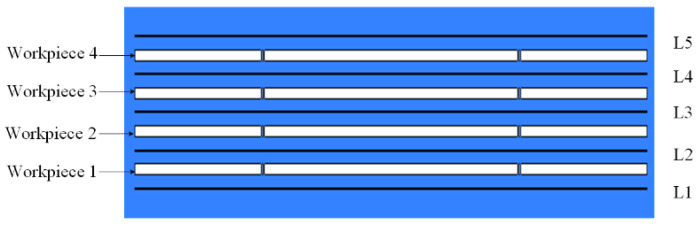
Line segment sampling diagram.

During the simulation-based optimization of layer spacing, the assessment criteria included the absence of stagnant zones and the uniformity of electrolyte flow velocity among the layers. To quantitatively evaluate the interlayer flow consistency, the electrolyte flow rates were analyzed along five 20 mm wire segments (L1–L5), as shown in Figure 5.

**Figure 6:**
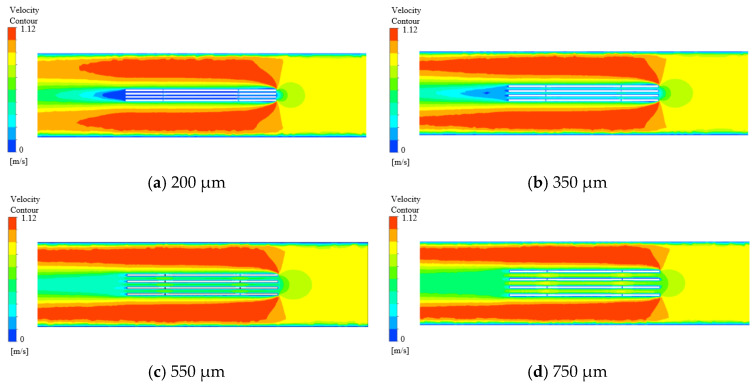
Contours of velocity magnitude.

**Figure 7:**
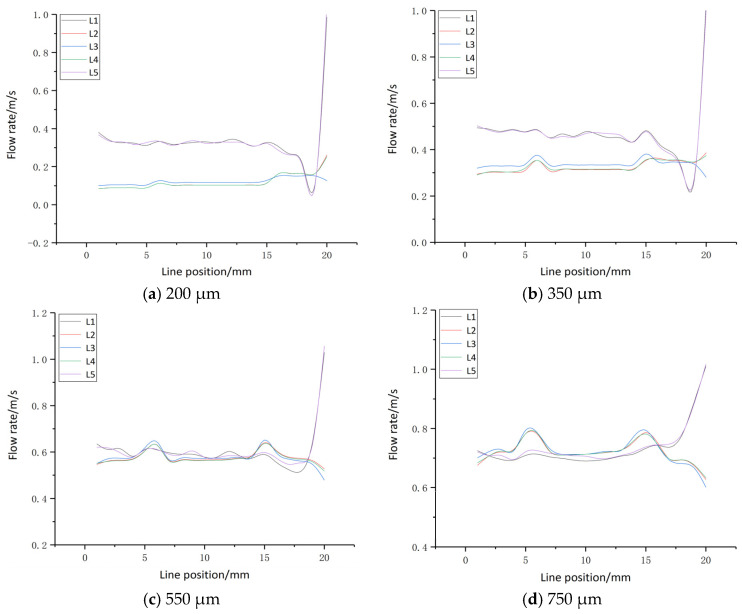
Flow rate curve.

The k-ε turbulence model was employed in the simulations. Gravitational effects were considered significant and were incorporated by specifying the gravitational acceleration direction along the Y-axis with a magnitude of 9.8 m/s^2^. An outflow boundary condition was applied at the outlet, while comprehensive details of the remaining simulation parameters are listed in Table 2. Analysis of the electrolyte flow dynamics within the interlayer regions was conducted across gap distances of 200 µm, 350 µm, 550 µm, and 750 µm. The resultant flow patterns are presented in Figure 6, and the corresponding velocity profiles across various gap sizes are illustrated in Figure 7.

As illustrated in Figure 6, under smaller inter-electrode gaps (200 µm and 350 µm), the electrolyte flow velocity on the left side of the workpiece is significantly reduced, resulting in the development of a stagnant region, commonly referred to as a “dead zone.” This indicates that although electrolyte entering from the nozzle penetrates the interlayer channels, the flow velocity therein remains insufficient. The bubbles and corrosion products generated during processing are difficult to quickly discharge and accumulate in the gaps, leading to secondary electrolysis, short circuits, or decreased processing accuracy. As a result, accumulated hydrogen bubbles and electrolytic by-products are not effectively expelled from the interlayer region. Hence, it is necessary to increase the workpiece gap appropriately when the inlet velocity is held constant. In contrast, at larger gaps of 550 µm and 750 µm, the electrolyte velocity on the left side of the workpiece increases significantly, eliminating the dead zone and enabling efficient removal of electrolysis products. This enhancement contributes to improved mass transport efficiency. The velocity profiles depicted in Figure 7 further demonstrate that at a gap of 550 µm, the electrolyte flow exhibits better uniformity across different layers. Therefore, a gap size of 550 µm is identified as the optimal configuration.

**Figure 8:**
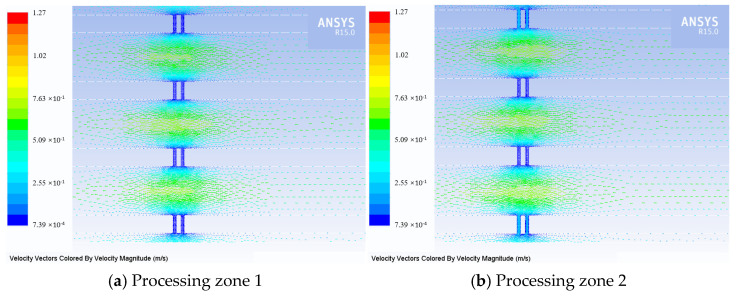
Velocity vectors diagram.

When the workpiece spacing is set at 550 µm, the flow behavior of the electrolyte within the processing zone is illustrated in Figure 8. As shown, a portion of the electrolyte entering the interlayer from the nozzle passes through the machining gap. The implemented flow serves to evacuate gaseous bubbles and reaction by-products, promotes electrolyte refreshment, and thereby enhances machining efficiency. Processing zone 1, being located farther from the flushing port compared to processing zone 2, exhibits a lower flow rate within the machining gap.

### 3.2. Micro Slit Processing Experiment

**Figure 9:**
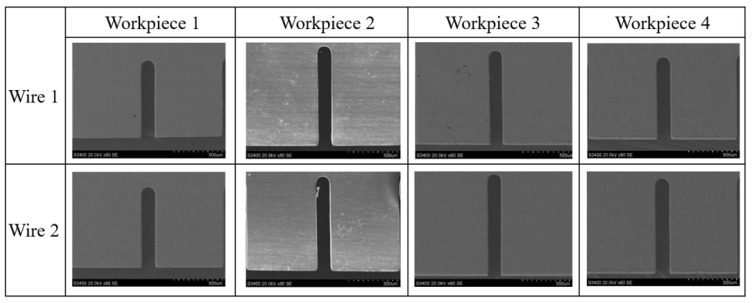
SEM images of the slits.

In this study, a pair of wire electrodes were employed to machine four-layer workpieces. The effects of critical process parameters on machining performance were investigated using a single-factor experimental approach. The parameters under study included feed rate, voltage, pulse frequency, and duty cycle. The element mass fractions (wt.%) of 3J53 constant modular alloy are: 42 Ni, 5.3 Cr, 2.5% Ti, 0.05 C, 0.8 Mn, 0.8 S, 0.02 P, 0.02 S, and the rest are Fe. A series of slots were fabricated on a 3J53 constant modulus alloy under the conditions specified in Table 3. Figure 9 characterizes the morphology of the machined slots. Subsequently, to quantitatively assess the impact of the process parameters on the slot dimensions, five separate measurements were taken at distinct locations along the length of each slot. The measuring equipment is a KEYENCE VHX-6000 series digital microscope (KEYENCE, Osaka, Japan), and the manufacturer’s trademark claims a measurement uncertainty of ±0.5 μm. The average of these five values was regarded as the width of the individual slot, while the mean of all such averages provided the overall average slot width. The standard deviation of the slot widths was used to evaluate machining consistency, with a smaller standard deviation indicating higher uniformity.

**Figure 10:**
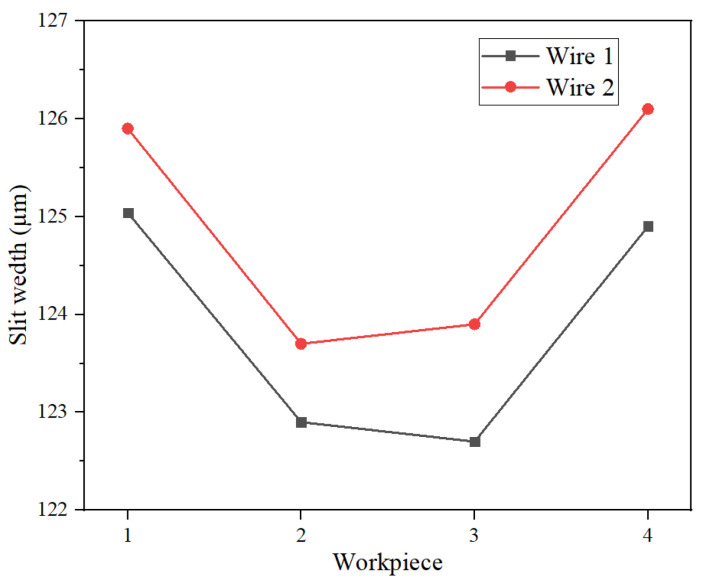
The variation law of slit width of each layer of workpiece with the position of the wire electrode.

The slit width measurements are plotted in Figure 10. It can be observed that, for the same workpiece, slots machined with wire electrode 2 exhibited greater widths than those produced with wire electrode 1. This discrepancy is attributed to the closer proximity of wire electrode 2 to the lateral fluid nozzle. This elevated flow in the interelectrode region of wire 2 served to enhance mass transfer efficiency, which accelerated the rate of the underlying electrochemical reactions. Consequently, the slot width obtained with wire electrode 2 was slightly larger.

## 4. Discussion

### 4.1. Effects of Feed Rate

**Figure 11:**
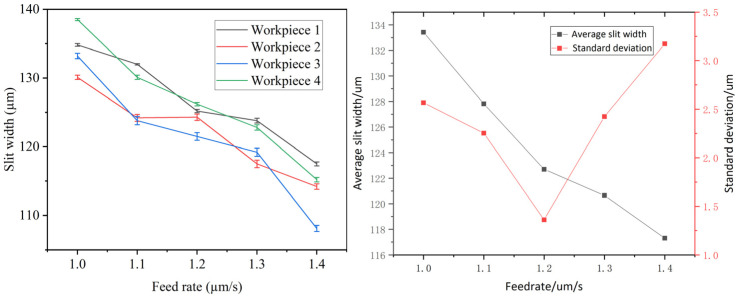
Feed rate versus average slit width and standard deviation of slit width.

The machining parameters were configured with an applied voltage of 10 V, a pulse frequency of 50 kHz, a duty cycle of 50%, and a feed rate varying between 1.0 and 1.4 µm/s. As shown in Figure 11, the average slit width and its standard deviation are presented. The results indicate that higher feed rates lead to a reduction in the average slit width. Conversely, the standard deviation first decreases and subsequently increases with rising feed rate. A higher feed rate reduces the residence time of the wire electrode at any given point along the machining path, resulting in reduced material removal and narrower slit widths. The minimum standard deviation is observed at a feed rate of 1.2 µm/s. When the feed rate falls below this threshold, the reduced speed of the electrode results in extended localized exposure, which enhances stray corrosion and diminishes machining precision. As the feed rate increases to the optimal value of 1.2 µm/s, the extent of stray corrosion is reduced, and machining precision is enhanced. However, once the feed rate surpasses this threshold, the slit width becomes detrimentally narrow. This restricts the efficient removal of electrolysis byproducts to the interlayer and limits the replenishment of fresh electrolyte. As a result, localized variations in electrolyte concentration within the gap promote inhomogeneous slit width formation and an elevated standard deviation.

### 4.2. Effects of Applied Voltage

**Figure 12:**
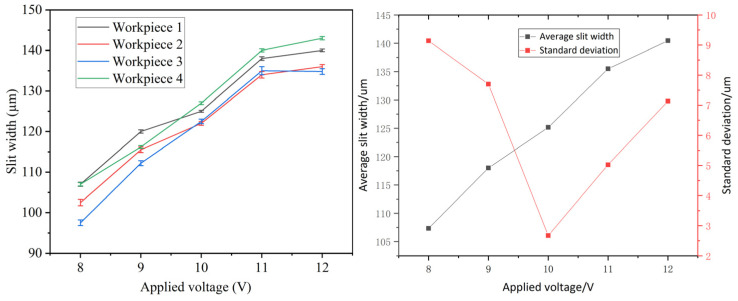
Variation in average slit width and standard deviation with applied voltage.

The processing parameters were set at a pulse frequency of 50 kHz, a duty cycle of 50%, and a feed rate of 1.2 µm/s, with the applied voltage ranging from 8 to 12 V. As illustrated in Figure 12, the average slit width exhibits an increasing trend with higher voltage, while the standard deviation initially decreases before rising. Elevated applied voltage enhances the current density in the machining region, which accelerates electrochemical reactions and increases the rate of anodic dissolution per unit time, resulting in wider slits. The smallest standard deviation occurs at 10 V. Below this voltage, low current densities result in narrow slit widths, which hinder the removal of electrolysis products and restrict electrolyte flow. This leads to significant variations in electrolyte concentration, reduced conductivity, non-uniform electric field distribution, and poorer slit consistency. Short-circuiting occurs below 8 V. Above 10 V, an increase in current density will reduce locality, exacerbate stray corrosion, and cause inconsistency in narrow slits. In addition, under high voltage, the reaction rate of hydrogen evolution at the cathode and oxygen evolution at the anode increases sharply, producing a large number of bubbles. Uneven distribution of electrolyte conductivity and unstable flow field result in random and uneven distribution of current density, thereby reducing machining shape accuracy and surface quality, and increasing the standard deviation of seam width.

### 4.3. Effects of Pulse Frequency

**Figure 13:**
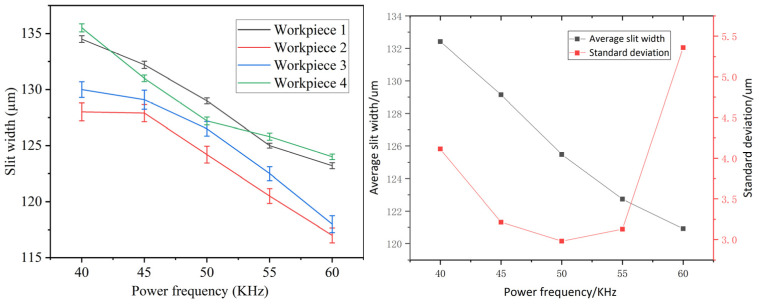
Relation between mean slit width, standard deviation of slit width and pulse frequency.

The machining parameters were configured as follows: an applied voltage of 10 V, a duty cycle of 50%, a feed rate of 1.2 µm/s, and a pulse frequency varying between 40 and 60 kHz. Figure 13 illustrates the trends in both the mean and standard deviation of the machined slit width. The results indicate that the mean slit width decreases gradually as the pulse frequency rises. In contrast, the standard deviation initially declines before increasing, with its minimum value—corresponding to the highest level of slit width uniformity—observed at a pulse frequency of 50 kHz. Higher pulse frequencies result in shorter pulse periods and, under a constant duty cycle, reduced pulse width. This leads to decreased anode dissolution of the workpiece and diminished electrolytic erosion capability of the cathode tool per pulse period, thereby reducing the slit width. At pulse frequencies below 50 kHz, the amount of electrolytic products generated per unit time decreases as the frequency rises. These products can be efficiently eliminated from the machining gap, thereby enhancing process stability and yielding a lower standard deviation in slit width. In contrast, at frequencies above 50 kHz, the slit width is reduced, which restricts electrolyte flow into the machining gap and compromises the evacuation of electrolytic by-products. This leads to inefficient mass transfer, substantial variations in electrolyte concentration within the gap, and consequently, an increase in the standard deviation of the slit width.

### 4.4. Effects of Duty Cycle

**Figure 14:**
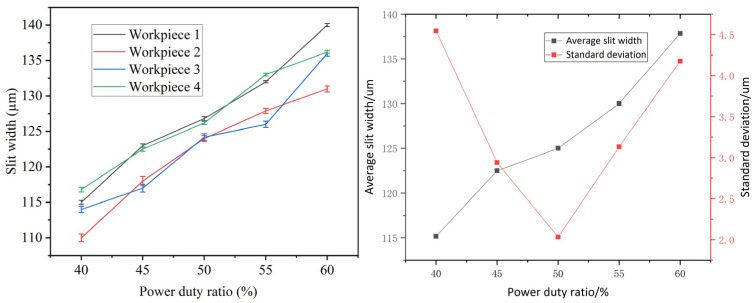
Relation between mean slit width, standard deviation of slit width and duty cycle.

The machining parameters were configured as follows: an applied voltage of 10 V, a pulse frequency of 50 kHz, a feed rate of 1.2 µm/s, and a duty cycle varying between 40% and 60%. As shown in Figure 14, both the mean and standard deviation of the machined slit width vary with the duty cycle. Specifically, the mean slit width exhibits a positive correlation with the duty cycle, while the standard deviation demonstrates an initial decrease followed by an increase. Under a fixed frequency condition, a higher duty cycle is associated with a broader pulse width, leading to an extended pulse-on time per cycle. This prolongs the duration of voltage application per period, leading to increased material removal from the anode workpiece, and consequently, a wider slit. The smallest standard deviation in slit width—indicating optimal consistency—occurs at a 50% duty cycle. The restricted aperture at duty cycles under 50% adversely affects the expulsion of electrolysis products, thereby having a detrimental effect on overall mass transfer efficiency. Additionally, the restricted gap promotes the growth and subsequent rupture of gas bubbles, which can induce vibration in the wire electrode and result in unstable machining conditions, thereby impairing slit width uniformity. Conversely, when the duty cycle exceeds 50%, the enhanced electrochemical dissolution capacity of the wire electrode generates more electrolysis products. Although the slit is wider, it remains insufficient for timely product removal. The reduced efficiency of debris expulsion and significant fluctuations in electrolyte concentration cause instability in the dissolution rate, leading to increased variability in the slit width.

### 4.5. Effects of Electrolyte Concentration

**Figure 15:**
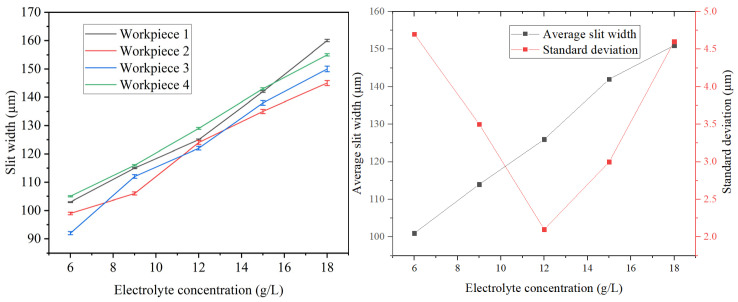
Relation between mean slit width, standard deviation of slit width and electrolyte concentration.

The machining parameters were configured as follows: an applied voltage of 10 V, a duty cycle of 50%, a feed rate of 1.2 μm/s, a pulse frequency of 50 kHz, and an electrolyte concentration ranging from 6 to 18 g/L. As shown in Figure 15, the mean and standard deviation of the machined slit width are illustrated. The overall average slit width exhibited an increasing trend with elevated NaNO_3_ concentration. This trend is attributed to the direct influence of electrolyte concentration on conductivity. As concentration rises, the enhanced conductivity accelerates the electrochemical reaction, thereby increasing the material removal rate per unit time. Consequently, the width of individual slits enlarges, leading to a greater mean slit width both per layer and overall. The reduction in the standard deviation of slit width with increasing electrolyte concentration was followed by an increase beyond 12 g/L, the optimum for consistency. At concentrations below 12 g/L, the low electrolyte concentration results in a slow reaction rate and narrower slits. This hinders the effective removal of electrolytic products by the wire electrode, reduces mass transfer efficiency, causes fluctuations in local electrolyte concentration, and impairs machining accuracy—all contributing to poor slit uniformity. At 6 g/L, intermittent short circuits and unstable machining behavior were observed, further exacerbating inconsistency. When the concentration surpassed 12 g/L, the further rise in conductivity led to higher current density within the machining zone, thereby amplifying stray corrosion and diminishing localization accuracy. Consequently, the standard deviation of the slit width increased, accompanied by a decline in slit uniformity.

### 4.6. Microstructure Fabrication

**Figure 16:**
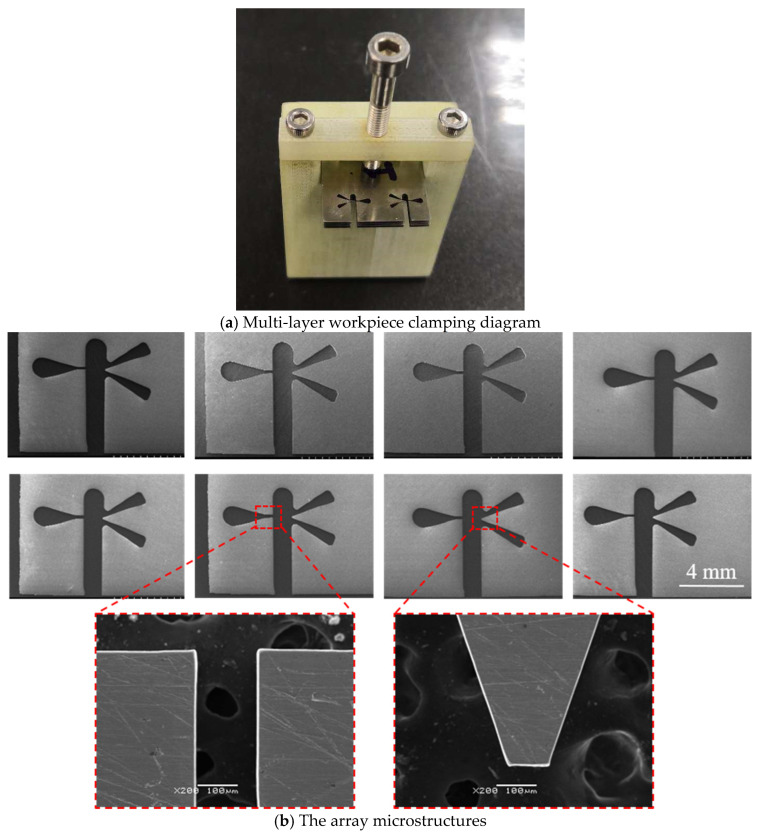
(**a**) Multi-layer workpiece mounting diagram and (**b**) array microstructure.

Based on the aforementioned experimental outcomes, the following processing parameters were established: two wire electrodes were utilized to process four workpieces, with an inter-workpiece distance of 550 µm. A NaNO_3_ electrolyte solution at a concentration of 12 g/L was employed, along with a supply voltage of 10 V. The pulsed power supply was configured to operate at 50 kHz with a 50% duty cycle, while the feed rate was maintained at 1.2 µm/s. Under these conditions, an arrayed microstructure (Figure 16) was successfully fabricated using a 200 µm-thick nickel-based super-elastic alloy. The overall machining speed achieved was 9.6 µm/s, reflecting a significant enhancement in processing efficiency. The complete process was completed in seven hours, demonstrating the effectiveness of the proposed method.

## 5. Conclusions

In this study, the horizontal electrolyte flushing method was used to improve the mass transfer efficiency in electrochemical micro machining (MWECM) of multi-wire and multi-layer workpieces, and the optimal process parameters were determined by combining flow field simulation and experimental research. The main findings are summarized as follows:

1. A multi-wire electrochemical microfabrication method under horizontal electrolytic flushing was proposed. The spacing between workpieces was optimized through fluid simulation based on ANSYS, and the optimal value was determined to be 550 µm.
2. Using the lateral flushing method, the influence of main process parameters on the consistency of slit width was studied. The variables tested include feed rate, voltage, frequency, duty cycle, and electrolyte concentration. Finally, the optimal processing parameters were determined.
3. Utilizing the optimized parameters, specifically a feed rate of 1.2 µm/s, arrayed microstructures were successfully fabricated, achieving an overall processing rate of 9.6 µm/s. The results demonstrate that MWECM significantly enhances machining efficiency compared to conventional wire electrochemical micromachining (WECM).

## Figures and Tables

**Table 1:** Flow field model parameters.

Parameter	Value
Thickness of the gasket (µm)	200, 350, 550, 750
Thickness of the workpiece (µm)	200
Length of the workpiece (mm)	20
Wire electrode diameter (µm)	50
Slit width (µm)	130

**Table 2:** Simulation parameters.

Parameter	Value
Inlet velocity (m/s)	0.8
Electrolyte density (Kg/m^3^)	1000
Electrolyte viscosity (Pa×s)	10^−3^

**Table 3:** Slit processing parameters.

Parameter	Value
Workpiece spacing	550 µm
Workpiece	200 µm thickness, 3J53 constant elastic alloy
Electrolyte	12 g/L, NaNO_3_ aqueous solution
Wire electrode diameter	50 µm
The inlet velocity of electrolyte nozzle	0.8 m/s
Amplitude	0.6 mm
Vibration frequency	2 Hz
Voltage amplitude	10 V
Duty cycle	50%
Pulse frequency	50 KHz
Feed rate	1.2 µm/s

## Data Availability

The original contributions presented in this study are included in the article. Further inquiries can be directed to the corresponding author.

## Abbreviations

The following abbreviations are used in this manuscript:

MWECM	Multi-layer workpieces and multiple wires electrochemical micromachining
MWECMF	Multi-layer workpieces and multiple wires electrochemical micromachining with horizontal electrolyte flushing
WECM	Wire electrochemical micromachining
